# Synthesis and Biological Evaluation of Novel (thio)semicarbazone-Based Benzimidazoles as Antiviral Agents against Human Respiratory Viruses

**DOI:** 10.3390/molecules25071487

**Published:** 2020-03-25

**Authors:** Valeria Francesconi, Elena Cichero, Silvia Schenone, Lieve Naesens, Michele Tonelli

**Affiliations:** 1 Dipartimento di Farmacia, Università di Genova, Viale Benedetto XV 3, 16132 Genova, Italy; francesconi.phd@difar.unige.it (V.F.); cichero@difar.unige.it (E.C.); schenone@difar.unige.it (S.S.); 2 Rega Institute for Medical Research, KU Leuven, Herestraat 49, B-3000 Leuven, Belgium; lieve.naesens@kuleuven.be

**Keywords:** (thio)semicarbazone-based benzimidazoles, hydrazone-based benzimidazoles, anti-RSV activity, anti-influenza activity, anti-coronavirus activity, molecular modelling studies

## Abstract

Respiratory RNA viruses are responsible for recurrent acute respiratory illnesses that still represent a major medical need. Previously we developed a large variety of benzimidazole derivatives able to inhibit these viruses. Herein, two series of (thio)semicarbazone- and hydrazone-based benzimidazoles have been explored, by derivatizing 5-acetyl benzimidazoles previously reported by us, thereby evaluating the influence of the modification on the antiviral activity. Compounds **6**, **8**, **16** and **17**, bearing the 5-(thio)semicarbazone and 5-hydrazone functionalities in combination with the 2-benzyl ring on the benzimidazole core structure, acted as dual inhibitors of influenza A virus and human coronavirus. For respiratory syncytial virus (RSV), activity is limited to the 5-thiosemicarbazone (**25**) and 5-hydrazone (**22**) compounds carrying the 2-[(benzotriazol-1/2-yl)methyl]benzimidazole scaffold. These molecules proved to be the most effective antiviral agents, able to reach the potency profile of the licensed drug ribavirin. The molecular docking analysis explained the SAR of these compounds around their binding mode to the target RSV F protein, revealing the key contacts for further assessment. The herein-investigated benzimidazole-based derivatives may represent valuable hit compounds, deserving subsequent structural improvements towards more efficient antiviral agents for the treatment of pathologies caused by these human respiratory viruses.

## 1. Introduction

**Figure 1:**
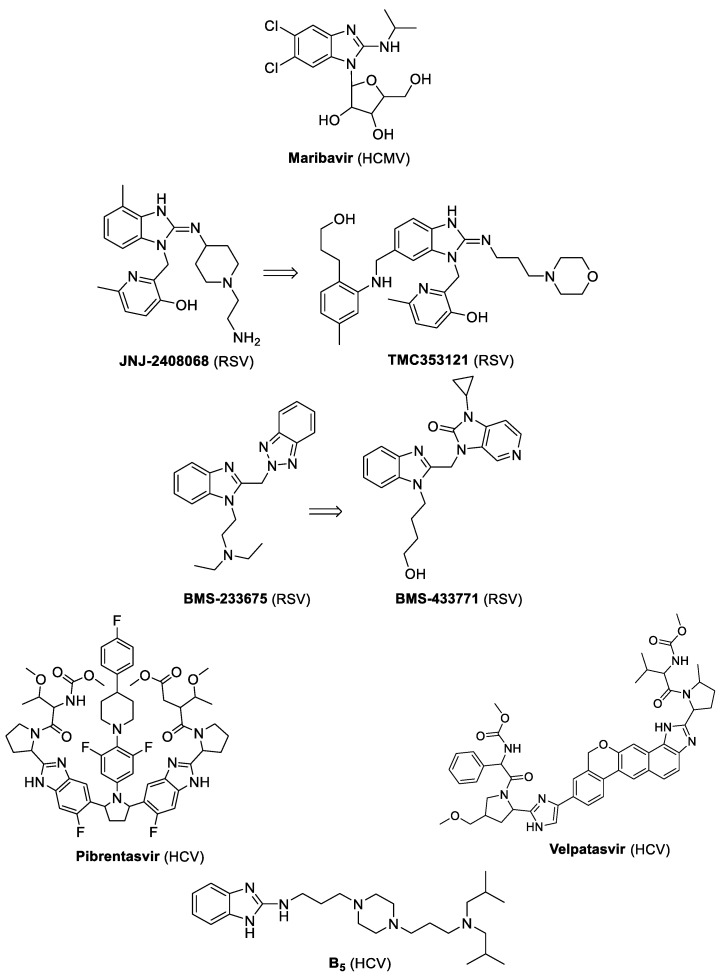
Some prototypic benzimidazole derivatives with activity against distinct viruses (mentioned between brackets).

The benzimidazole scaffold is a structural isostere of naturally occurring nucleobases. Many approved drugs bear a benzimidazole ring as their main unit or important substructure [1,2]. Benzimidazole-based agents are also considered for antimicrobial purposes, such as antituberculosis, antiprotozoan or antiviral activities [3,4]. Maribavir, a benzimidazole riboside with strong activity against cytomegalovirus (HCMV), is undergoing Phase III evaluation (clinicaltrials.gov NCT02927067 and NCT02931539) (Figure 1). Different substitutions on the benzimidazole nucleus of maribavir were reported to entail activity against flaviviruses, HIV, hepatitis B virus, hepatitis C virus (HCV) or respiratory syncytial virus (RSV) [5]. Besides, a few non-nucleoside benzimidazole derivatives interfering with RSV F protein-mediated fusion, namely, JNJ-2408068 (R-170591), BMS-433771 and TMC353121, have reached a far stage of (pre)clinical development, but have been halted after a negative outcome [6,7] (Figure 1). Two FDA-approved inhibitors of the HCV NS5A protein, pibrentasvir and velpatasvir, contain a benzimidazole nucleus (Figure 1). The benzimidazole derivative B5, under clinical evaluation for the treatment of neurodegenerative diseases, was shown to delay HCV infection in a humanized mouse model [8] (Figure 1). The literature further contains examples of other benzimidazole derivatives with activity against unrelated viruses, such as influenza virus or poliovirus.

Another chemical entity with relevance for antimicrobial drug development is the (thio)semicarbazone moiety. Compounds containing this structure may interfere with processes that are essential for microbe survival, such as deoxyribonucleotide synthesis, bacterial cell wall synthesis or maintenance of thiol content [9,10]. Interest in their antiviral properties dates back to the discovery of methysazone (Marboran^®^), which was used to treat smallpox prior to its global eradication [11]. More recent studies have reported activity against other poxviruses, herpes simplex virus or influenza virus [12]. Transformation of some 5-acetyl-2-phenylbenzimidazoles into the corresponding (thio)semicarbazone analogues yielded new agents with activity against HCV and the related bovine viral diarrhea virus (BVDV) [13]. Inhibition of the BVDV RNA polymerase was reported for 5,6-dimethoxy-1-indanone-derived thiosemicarbazones [14].

**Figure 2:**
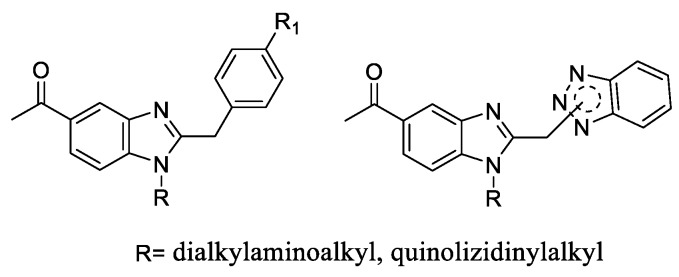
Scaffold structures of our Series 1 (5-acetyl substituted 2-benzylbenzimidazoles) and Series 2 (2-[(benzotriazol-1/2-yl)methyl]benzimidazoles), which we previously reported as antiviral agents.

During the few last years, we synthesized a large variety of benzimidazole-based derivatives and we explored their antitumor [15], analgesic [16] or antiviral potential. We focused on two series, i.e., 2-benzylbenzimidazoles (Series 1) [17] and 2-[(benzotriazol-1/2-yl)methyl)benzimidazoles (Series 2) [18,19] 1-substituted with a basic chain. Among them, we obtained analogues with promising activity against diverse RNA viruses (Figure 2).

In the light of our previous findings, we deemed it interesting to explore new additional substitution patterns on the benzimidazole main core, with the aim of gaining a better understanding of the potential of this nucleus towards the development of novel series of antiviral agents.

## 2. Results and Discussion

**Figure 3:**
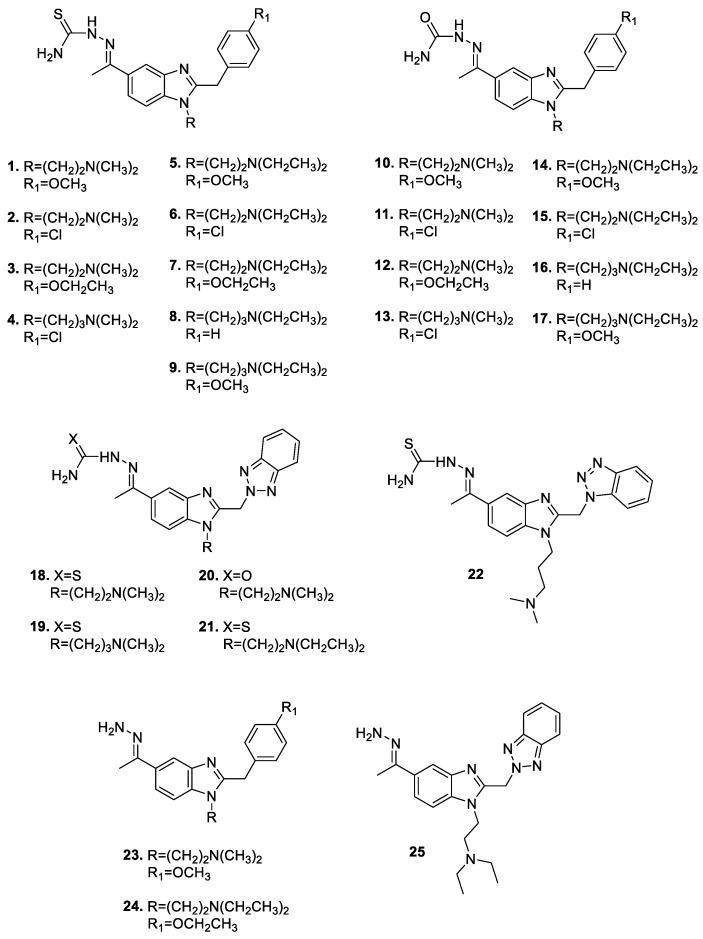
Chemical structures of the novel (thio)semicarbazone- and hydrazone-incorporating benzimidazoles (**1–25**).

Since the 5-acetyl substituted benzimidazoles were generally endowed with low efficacy, we explored alternative substitutions at position 5 of the benzimidazole ring, by synthesizing (thio)semicarbazone and hydrazone derivatives (Figure 3). These new compounds were evaluated for their activities against a broad panel of viruses and cytotoxicity in several mammalian cell lines. The main results are presented in Table 1 and Table 2, from which we omitted the compounds having no antiviral nor cytotoxic activity (at 100 µM, the highest concentration tested).

These investigations were accompanied by docking studies of the title compounds in complex with the RSV fusion F protein, which allowed the identification of the most relevant features involved in the protein-inhibitor recognition.

### 2.1. Chemistry

The starting 5-acetyl-2-benzylbenzimidazoles (Series 1) have been synthesized according to the literature [20], by the reaction of the proper 4-acetyl-1,2-phenylenediamine with the hydrochloride of the iminoester, previously obtained from the corresponding nitrile under Pinner conditions. The condensation at 180 °C of a mixture of the proper 1,2-phenylenediamine with the (benzotriazol-1/2-yl)acetic acid has given the 5-acetyl-2-[(benzotriazol-1/2-yl)methyl]benzimidazoles [21].

**Scheme 1:**
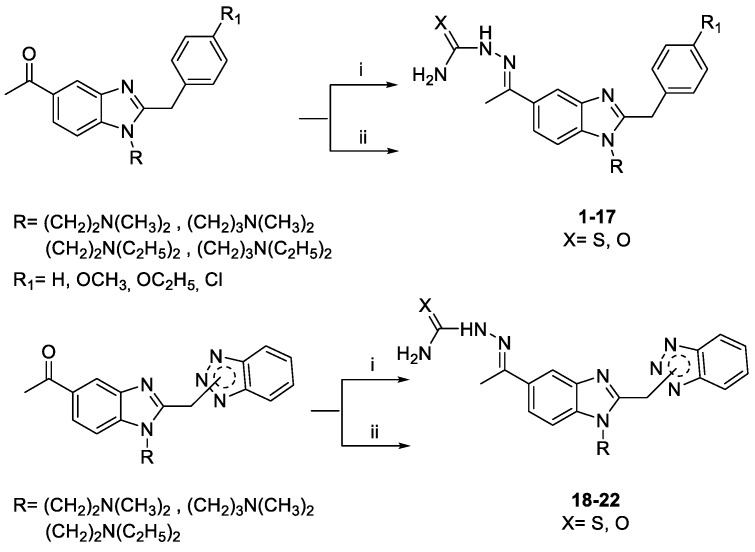
Reagents and conditions: (i) thiosemicarbazide in EtOH, H_2_O and glacial CH_3_COOH, 3 h at reflux; (ii) semicarbazide ·HCl in EtOH and CH_3_COONa 1N, 4 h at reflux.

**Scheme 2:**
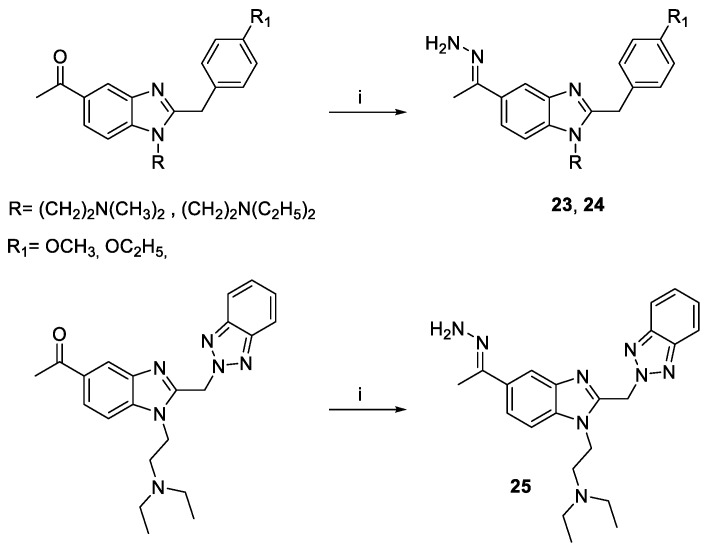
Reagents and conditions: (i) EtOH/H_2_O, NH_2_NH_2_ · H_2_O (1.15 equiv.), 120 °C, 5 h.3.

Our target compounds **1**–**25** have been obtained following the synthetic routes presented in Scheme 1 and Scheme 2. Thiosemicarbazones **1**–**9**, **18**, **19**, **21** and **22**, and semicarbazones **10**–**17** and **20** have been prepared by refluxing an ethanolic/aqueous solution of thiosemicarbazide or semicarbazide hydrochloride in the presence of glacial acetic acid or sodium acetate, respectively.

The reaction at reflux of the proper 5-acetyl benzimidazole with a slight excess of hydrazine hydrate for 5 h has afforded the target hydrazone derivatives **23**–**25** (Scheme 2). The structures of the novel compounds have been confirmed using ^1^H and ^13^C NMR, and elemental analysis. The purity of compounds (checked by elemental analysis) has been in all cases >95%.

Thiosemicarbazones are known to display thione-thiol tautomerism, since they contain the hydrazidic proton (-C(=S)NH-N=) that can shift onto the sulfur atom, leading to a thiol form stabilized by conjugation (-C(-SH)=N-N=). Indeed, on the 1H NMR spectra our compounds do not exhibit the signal at 4.00 ppm, attributable to –SH proton, suggesting that the thione form is the only tautomer. The Schiff base of our thiosemicarbazone moiety acquires E isomerism since the hydrazinic proton (-C(=S)NH-N=) falls in the 9–12 ppm range [22]. In fact this signal appears at ca 10.18 ppm, whilst the thioamide protons (-C(=S)NH_2_) exhibit two different chemical shifts at ca 8.25 ppm and 7.94 ppm. The same consideration can be made for semicarbazones with the exception of the signal of amide group (CONH_2_) which appears as a broad singlet (ca 6.50 ppm) with the two protons being indistinguishable. The different behaviors of the two series may be explained by the restricted free rotation brought about by the formation of the carbon–nitrogen double bond character relative to thione-thiol tautomerism [23]. In addition, it is noteworthy that the sulfur atom of the thione tautomer has a greater atomic radius than oxygen atom of the corresponding semicarbazone, thereby making the thioamide protons magnetically different for steric hindrance.

### 2.2. Biological Evaluation SAR

As explained above, the new compounds were synthesized from 5-acetyl substituted 2-benzylbenzimidazoles and 2-[(benzotriazol-1/2-yl)methyl]benzimidazoles (Series 1 and 2; Figure 2). We previously published that Series 2 has high potential against RSV, with the best performing derivatives having nanomolar activity [18,19]. These compounds carried at position 1 of the benzimidazole ring different basic chains, such as the most efficacious quinolizidinylalkyl [(octahydro-2H-quinolizin-1-yl)alkyl] chains or the dialkylaminoalkyl ones [18,19].

The new compounds (**1**–**25**) were evaluated against a broad panel of RNA and DNA viruses in suitable mammalian cell culture assays [24,25,26,27]. The antiviral activity data obtained by microscopic inspection of the viral CPE (data not shown) were in agreement with those obtained by the colorimetric MTS cell viability test (Table 1). The most active compounds **22** and **25** exhibited EC_50_ values of 7.0 and 2.4 µM in the MTS method, *versus* 8.9 and 2.4 µM in the microscopic method.

Seven compounds displayed activity against one of more human respiratory viruses; i.e., RSV (**22** and **25**), influenza A virus (**6**, **8**, **16**, **17** and **22**) or human coronavirus (**6**, **8**, **16**, **17** and **24**) (Table 1). Compounds **22** and **25** had anti-RSV EC_50_ values of 7.0 and 2.4 µM, respectively, which makes them equipotent to the reference drug ribavirin (EC_50_ of 6.7 µM). A lower level of activity was seen for influenza A and coronavirus, with the EC_50_ values falling in the range of 25–86 µM. Despite this relatively weak activity, it is relevant to note that compounds **6**, **8**, **16**, **17** and **24** are the first benzimidazole derivatives reported as active against coronavirus.

The following careful SAR analysis could be made. For RSV, activity is restricted to the 5-(thio)semicarbazone (**25**) and hydrazone (**22**) compounds carrying the 2-[(benzotriazol-1/2-yl)methyl]benzimidazole scaffold, in line with the previously synthesized analogues (see above), which show comparable potency in the low micromolar range. 

Regarding influenza A and coronaviruses, the activity is promoted by (thio)semicarbazone and hydrazone functionalities, especially when combined with the benzyl ring (**6**, **8**, **16**, **17** and **24**) compared to the bulkier (benzotriazol-1/2-yl)methyl skeleton (**22**). The nature of the substituent in the *para* position of the benzyl ring (H, Cl, OCH_3_) does not seem to have significant impact on the antiviral activity, since the unsubstituted derivatives (**8**, **16**; R_1_ = H) had comparable potency of those decorated with electron-withdrawing (**6**; R_1_ = Cl) or electron-donor groups (**17**; R_1_ = OCH_3_).

Finally, most compounds were devoid of cytotoxicity at 100 µM, the highest concentration tested. Two compounds, **17** and **24**, produced cytotoxicity in two of the four cell lines. The other molecules were either not toxic or exhibited a CC_50_ value of about 50 µM in one of the four cell lines. 

Interestingly, influenza A and human coronavirus shared sensitivity to the same inhibitors, **6**, **8**, **16** and **17**, whose definition of the mechanism of action is beyond the scope of this exploratory work. 

**Figure 4:**
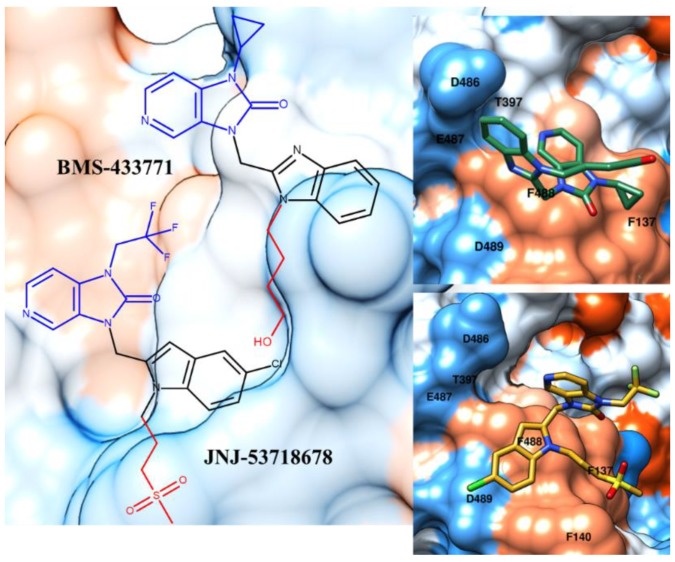
Chemical structure and X-ray positioning of BMS-433771 (pdb code: 5EA7) [31] and JNJ-53718678 (pdb code: 5KWW) [32] in complex with the RSV F protein. The chemical motifs of the two inhibitors featuring quite comparable contacts with the biological target are highlighted in blue and red. Hydrophobic and polar areas of the protein are represented as blue and orange regions on the RSV F protein’s Connolly surface.

As is well-known from literature, the antiviral activity against RSV is limited to several benzimidazole derivatives (Figure 1) [28,29], and also to the more recent analogue JNJ-53718678 (Figure 4) [30], which were demonstrated to impair the viral replication machinery by blocking the F protein-induced membrane fusion. From 2017, JNJ-53718678 entered Phase 2 studies in adults and infants for therapy of RSV infections (ClinicalTrials.gov Identifier NCT03379675, NCT03656510, NCT04056611). Due to the substantial structural similarity between the newly synthesized compounds and the above anti-RSV (pre)clinical candidates, molecular modeling studies were performed in order to reveal the most important features underlying the F protein/ligand interactions.

### 2.3. Molecular Modelling Studies

During the last few years, a number of crystallographic structures of the prefusion RSV glycoprotein became available focusing on several benzimidazole-based or bioisosteres inhibitors as co-crystallized ligands [31,32,33]. A number of them highlighted a small number of contacts responsible for the inhibitor positioning at the exposed surface area of the protein, occupying one hydrophobic region of the biological target especially, including the F137 residue. As shown in Figure 4, the inhibitors BMS-433771 and JNJ-53718678 move the 1,3-dihydroimidazo[4,5-c]pyridin-2-one ring into the proximity of the aforementioned F137 and F488 amino acids, detecting the first one π-π stacking with these residues. The substitution of the alkoxy chain of BMS-433771 with the JNJ-53718678 sulfone moiety makes the compound more hydrophobic than the prototype, flatting the JNJ-53718678 indole core towards F137, F140 and F488.

**Figure 5:**
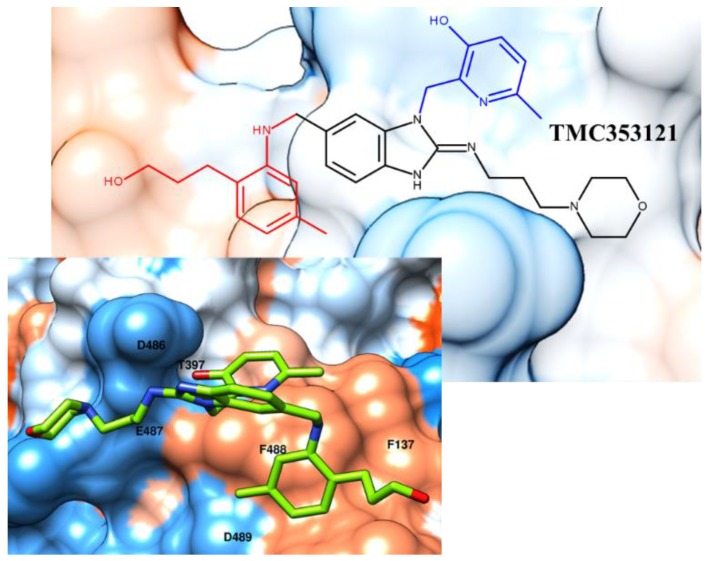
Chemical structure and X-ray positioning of TMC353121 (pdb code: 5EA5) [31] in complex with the RSV F protein. The chemical motif of the inhibitor maintained the key contacts displayed by the analogues BMS-433771 and JNJ-53718678; they are highlighted in blue and red. Hydrophobic and polar areas of the protein are represented as blue and orange regions on the RSV F protein’s Connolly surface.

Interestingly, the presence of the alkyl-morpholine chain linked to the dihydrobenzimidazole core of the compound TMC353121 allowed us to explore another RSV F protein binding pocket, which is able to anchor the inhibitor at the exposed surface of the protein (see Figure 5).

Indeed, the X-ray crystallographic data of this ligand in complex with the F protein reveal polar contacts between the TMC353121 protonated nitrogen atom of the morpholine ring and a polar area of the protein, including D486 and E487. On the other hand, the pyridine group and the terminal phenyl ring were properly oriented toward T397, detecting one H-bond with the biological target, and around the hydrophobic pocket previously discussed for BMS-433771 and JNJ-53718678 characterized by F137 and F488. On this basis, it is thought that interactions with F137 are mandatory to exert RSV F protein inhibition; the most promising compounds are better stabilized at the surface of the protein by additional H-bonds with the near polar pocket.

Herein we discuss this computational work with the aim of exploring an adequate RSV F protein inhibition activity of the newly synthesized antiviral compounds, by means of molecular docking simulations taking into account the X-ray crystallographic structure of the RSV F protein in complex with JNJ-53718678 (pdb code = 5KWW; resolution = 2.5 Å) [32]. This crystallographic data have been chosen based on the structural similarity shown by the reference compound JNJ-53718678 with the in-house, benzimidazole-based hydrazones and thiosemicarbazones. Currently, JNJ-53718678 is the more promising clinical candidate, whose potential therapeutic is undergoing evaluation in patients at high risk to developing acute RSV lower respiratory tract infections (ClinicalTrials.gov Identifier NCT03379675, NCT03656510, NCT04056611).

The main issues to be addressed were to clarify the role played by the hydrazone moiety and by the thiosemicarbazone at the position five of benzimidazole main core, especially when accompanied by a benzyl or a benzotriazole-1-yl or benzotriazole-2-yl ring linked at position 2 of the inhibitor scaffold. This was done through docking studies of all the newly synthesized compounds. According to our calculations (Table 3), only compounds **22** and **25** properly bind the exposed surface of the RSV F protein—they are anchored in the hydrophobic pocket by π-π stacking and cation-π interactions with F137, F140 and F488.

**Figure 6:**
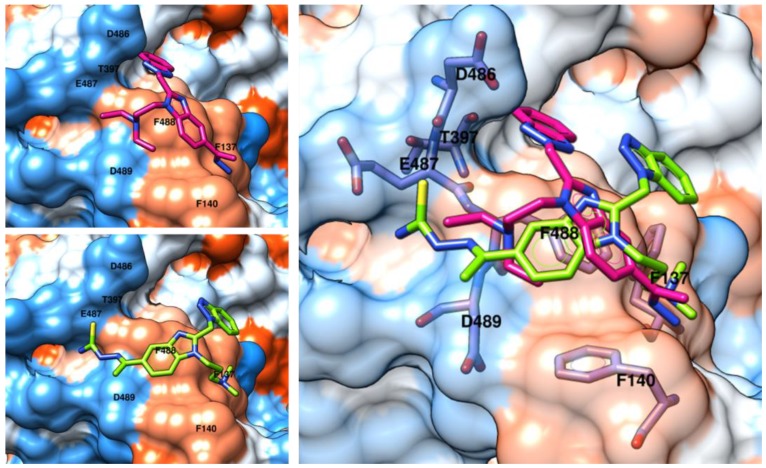
X-ray crystallographic data of the RSV F protein (pdb code: 5KWW; C atom; purple). Docking positioning of the inhibitors **22** and **25** (C atom; green and deep magenta, respectively) are shown within the surface of the protein (left side). Superimposition of the docking poses of **22** and **25** are shown on the right side. The most important residues are labelled and colored by atom type.

As shown in Figure 6, both the inhibitors moved the benzimidazole core in proximity of the aforementioned aromatic residues (π-π interactions) while the hydrazone group of **25** and the protonated amine chain of **22** were projected near F137, featuring cation-π stacking and H-bonds with the oxygen atom of the F137 carbonyl group.

Notably, the replacement of the hydrazone group of **25** with the thiosemicarbazone moiety of **22** switched the positioning of the inhibitor **22**, moving it the thiosemicarbazone moiety and the amine chain near the amine group and the hydrazone function of the analogue **25**; we detected comparable contacts with E437 and F137, respectively. Changing the benzotriazol-2-yl ring of **25** with the benzotriazol-1-yl one of **22** quite shifts the overall positioning of this portion of the molecule, even if it was oriented towards the same pocket. Interestingly, the benzotriazol-2-yl ring of **25** was H-bonded to the T397 side-chain.

While maintaining the hydrazone moiety in tandem with the substitution of the benzotriazolyl group with a decorated phenyl one led to the inactive compound **24**, unable to be stabilized at the surface of the protein, the replacement of the hydrazone group with the thiosemicarbazone one led to the analogue **21**. Interestingly, this featured H-bonds between the thiosemicarbazone function and E487, while the benzotriazolyl group and the amine chain moved far from the corresponding heteroaromatic group and hydrazone moiety of the analogue **25** (see Figure S1). On the other hand, even if this kind of positioning was in agreement with that previously discussed for the effective analogue **22**, it was unable to detect the same contacts displayed by **22** probably because of the different docking mode driven by the benzotriazol-1-yl skeleton in place of the benzotriazole-2-yl one of **25**. Accordingly, compound **19** exhibited a comparable docking mode with that of the analogue **21**, lacking the key contacts with F137 at the thiosemicarbazone moiety or amine chain group (Figure S2). Finally, the introduction of the smaller phenyl ring instead of the benzotriazolyl core at position two of the benzimidazole main ring (compound **4**) moved the amine chain towards the protein’s deep crevice delimited by T397 while the phenyl moiety detected π-π stacking with F137, missing any H-bonds with this residue. Conversely, the thiosemicarbazone group was H-bonded to E487 (see Figure S3). Accordingly, this compound was poorly effective if compared with the analogue **22**.

In this work we also evaluated, by computational prediction, a number of descriptors related to absorption, distribution, metabolism and excretion properties (ADME). This represents a useful in silico strategy for accelerating the discovery of drug-like compounds [33]. For the most promising antiviral compounds, **22** and **25**, we calculated the number of H-bonding acceptor and donor groups and rotable bonds, the logarithmic ratio of the octanol-water partitioning coefficient (cLogP), the human intestinal absorption (HIA), the volume of distribution (Vd), the binding to plasmatic proteins (%PPB) and albumin (LogKa HSA) and the oral bioavailability percentage (%F). As shown in Table 4, all the compounds were characterized by a favorable profile in terms of lipophilicity—that being an LogP below 5 (Lipinski rules)—and also displayed the ability to be fully adsorbed at the human intestinal membrane. With respect to the reference compound, **22** and **25** featured higher oral bioavailability and lower binding to plasmatic proteins.

Docking studies allowed us to rationalize the SAR observed in these anti-RSV benzimidazoles and to recognize the compounds **25** and **22** as the best suited F protein inhibitors. The preliminary information concerning their pharmacokinetic properties pointed to a favorable drug-like profile, enabling these anti-RSV agents to undergo a further optimization process.

## 3. Materials and Methods

### 3.1. Chemistry

#### 3.1.1. General Information

Chemicals, solvents and reagents were used as obtained from commercial sources (Alfa Aesar and Sigma-Aldrich) without further purification. Column chromatography (CC): silica gel (SiO_2_) (Merck). Mps: Büchi apparatus, uncorrected. ^1^H NMR and ^13^C NMR spectra were recorded on Varian Gemini-200 spectrometer using DMSO-*d_6_* as a solvent. The chemical shifts (δ) in ppm were measured relative to tetramethylsilane (TMS). *J* in Hz. Elemental analyses were performed on Flash 2000 CHNS (Thermo Scientific) instrument in the Microanalysis Laboratory of the Department of Pharmacy, University of Genoa. Benz = benzimidazole ring; Bzt = benzotriazole ring; Arom = phenyl ring.

#### 3.1.2. General Procedure for the Preparation of Thiosemicarbazones

To a solution of the proper 5-acetyl benzimidazole (0.80 mmol) in ethanol (2 mL), a solution of thiosemicarbazide (0.85 mmol) in water (2.8 mL) and glacial acetic acid (0.22 mL) was added. The mixture was refluxed for 3h under stirring. The reaction mixture was then evaporated under vacuum, yielding an oily residue that was treated with warm water to get rid of the remaining thiosemicarbazide. The crude was purified by CC (SiO_2_, CH_2_Cl_2_ + 5% DEA), affording the final product as a white solid.

2-(1-{1-[2-(N,N-Dimethylamino)ethyl]-2-(4-methoxybenzyl)-1H-benzo[d]imidazol-5-yl}-ethylidene)hydrazine-1-carbothioamide (**1**): Yield: 36%; m.p. 183–84 °C. ^1^H NMR (200 MHz, DMSO-*d_6_*): 10.18 (s, 1H, NH), 8.24 (s, 1H, NH_2_), 8.16 (s, 1H, H(4)benz.), 8.00–7.83 (m, 1H, H(7)benz.), 7.98 (s superimposed, 1H, NH_2_), 7.44 (d, *J* = 8.6 Hz, 1H, H(6)benz.), 7.24 (d, *J* = 8.6 Hz, 2H, H(3′,5′)arom.), 6.90 (d, *J* = 8.6 Hz, 2H, H(2′,6′)arom.), 4.32–4.08 (m, 4H, CH_2_-Ar and CH_2_CH_2_-N(CH_3_)_2_), 3.74 (s, 3H, -OCH_3_), 2.38 (pseudo s, 5H, 3H CH_3_C=N- and 2H, CH_2_CH_2_-N(CH_3_)_2_), 2.13 (s, 6H, N(CH_3_)_2_). ^13^C-NMR (50 MHz, DMSO-*d_6_*): 178.13, 157.63, 154.48, 148.55, 141.98, 135.97, 131.00 (2C), 129.38 (2C), 128.22, 120.46, 116.96, 113.57 (2C), 109.37, 54.67, 45.00 (2C), 41.09, 31.88, 13.99. Analysis calculated for C_22_H_28_N_6_OS: % C 62.24, H 6.65, N 19.79, S 7.55; found: % C 62.44, H 6.67, N 19.80, S 7.16.

2-{1-[2-(4-Chlorobenzyl)-1-[2-(N,N-dimethylamino)ethyl]-1H-benzo[d]imidazol-5-yl]-ethylidene}hydrazine-1-carbothioamide (**2**): Yield: 42%; m.p. 199–200.5 °C. ^1^H NMR (200 MHz, DMSO-*d_6_*): 10.18 (s, 1H, NH), 8.24 (s, 1H, NH_2_), 8.12 (s, 1H, H(4)benz.), 7.97 (s superimposed, 1H, NH_2_), 7.93 (d superimposed, *J* = 9.2 Hz, 1H, H(7)benz.), 7.58–7.22 (m, 5H, H(6)benz. and H(2′,3′,5′,6′)arom.), 4.34 (s superimposed, 2H, CH_2_-Ar), 4.40–4.18 (m superimposed, 2H, CH_2_CH_2_-N(CH_3_)_2_), 2.37 (pseudo s, 5H, 3H CH_3_C=N- and 2H, CH_2_CH_2_-N(CH_3_)_2_), 2.13 (s, 6H, N(CH_3_)_2_). ^13^C-NMR (50 MHz, DMSO-*d_6_*): 178.12, 153.82, 148.54, 142.00, 135.72, 135.61, 131.08, 130.87, 130.42 (2C), 128.01 (2C), 120.91, 117.77, 109.82, 57.71, 45.06 (2C), 41.39, 32.01, 14.00. Anal. calcd. for C_21_H_25_ClN_6_S: % C 58.80, H 5.87, N 19.59, S 7.47; found: % C 58.68, H 5.57, N 19.82, S 7.11.

2-(1-{1-[2-(N,N-Dimethylamino)ethyl]-2-(4-ethoxybenzyl)-1H-benzo[d]imidazol-5-yl}-ethylidene)hydrazine-1-carbothioamide (**3**): Yield: 47%; m.p. 177–180 °C. ^1^H NMR (200 MHz, DMSO-*d_6_*): 10.18 (s, 1H, NH), 8.23 (s, 1H, NH_2_), 8.11 (s, 1H, H(4)benz.), 7.96 (s superimposed, 1H, NH_2_), 7.92 (d superimposed, *J* = 8.6 Hz, 1H, H(7)benz.), 7.45 (d, *J* = 8.6 Hz, 1H, H(6)benz.), 7.19 (d, *J* = 7.6 Hz, 2H, H(3′,5′)arom.), 6.87 (d, *J* = 8.0 Hz, 2H, H(2′,6′)arom.), 4.24 (pseudo s, 4H, CH_2_-Ar and CH_2_CH_2_-N(CH_3_)_2_), 3.98 (d, *J* = 6.2 Hz, 2H, OCH_2_CH_3_), 2.37 (pseudo s, 5H, 3H CH_3_C=N- and 2H, CH_2_CH_2_-N(CH_3_)_2_), 2.12 (s, 6H, N(CH_3_)_2_), 1.41–1.18 (m, 3H, OCH_2_CH_3_). ^13^C-NMR (50 MHz, DMSO-*d_6_*): 178.20, 156.87, 154.44, 148.58, 141.84, 135.79, 130.99 (2C), 129.36 (2C), 128.15, 120.42, 117.02, 114.04 (2C), 109.31, 62.54, 57.46, 45.03 (2C), 41.21, 31.91, 14.25, 14.00. Anal. calcd. for C_23_H_30_N_6_OS: % C 62.99, H 6.09, N 19.16, S 7.30; found: % C 63.12, H 6.48, N 19.16, S 7.07.

2-{1-[2-(4-Chlorobenzyl)-1-[3-(N,N-dimethylamino)propyl]-1H-benzo[d]imidazol-5-yl]-ethylidene}hydrazine-1-carbothioamide (**4**): Yield: 45%; m.p. 110–111 °C. ^1^H NMR (200 MHz, DMSO-*d_6_*): 10.18 (s, 1H, NH), 8.26 (s, 1H, NH_2_), 8.13 (s, 1H, H(4)benz.), 7.97 (s superimposed, 1H, NH_2_), 8.10–7.87 (m superimposed, 1H, H(7)benz.), 7.47 (d superimposed, *J* = 8.6 Hz, 1H, H(6)benz.), 7.37 (pseudo s superimposed, 4H, H(2′,3′,5′,6′)arom.), 4.35 (s superimposed, 2H, CH_2_-Ar), 4.20 (pseudo s superimposed, 2H, CH_2_CH_2_CH_2_-N(CH_3_)_2_), 2.37 (pseudo s, 3H, CH_3_C=N-), 2.07 (pseudo s, 8H, N(CH_3_)_2_ and CH_2_CH_2_CH_2_-N(CH_3_)_2_), 1.69 (pseudo s, 2H, CH_2_CH_2_CH_2_-N(CH_3_)_2_). ^13^C-NMR (50 MHz, DMSO-*d_6_*): 178.22, 153.66, 148.54, 141.88, 135.64 (2C), 131.08, 130.90, 130.32 (2C), 128.06 (2C), 120.54, 117.11, 109.33, 55.17, 44.58 (2C), 40.74, 31.80, 26.59, 14.00. Anal. calcd. for C_22_H_27_ClN_6_S: % C 59,65, H 6.14, N 18.97, S 7.24; found: % C 59.51, H 6.18, N 19.24, S 7.18.

*2-(1-{1-[2-(N,N-Diethylamino)ethyl]-2-(4-methoxybenzyl)-1H-benzo[d]imidazol-5-yl}-ethylidene)hydrazine-1-carbothioamide (**5**)*: Yield: 35%; m.p. 165–168 °C. ^1^H NMR (200 MHz, DMSO-*d_6_*): 10.16 (s, 1H, NH), 8.25 (s, 1H, NH_2_), 8.10 (s, 1H, H(4)benz.), 7.94 (s superimposed, 1H, NH_2_), 8.15-7.85 (m superimposed, 1H, H(7)benz.), 7.43 (d superimposed, *J* = 8.6 Hz, 1H, H(6)benz.), 7.20 (d, *J* = 8.2 Hz, 2H, H(3′,5′)arom.), 6.89 (d, *J* = 7.8 Hz, 2H, H(2′,6′)arom.), 4.26 (s superimposed, 2H, CH_2_-Ar), 4.17 (pseudo s superimposed, 2H, CH_2_CH_2_-N(CH_2_CH_3_)_2_), 3.72 (s, 3H, OCH_3_), 2.58–2.24 (m superimposed to DMSO, 6H, 2H CH_2_CH_2_-N(CH_2_CH_3_)_2_ and 4H CH_2_CH_2_-N(CH_2_CH_3_)_2_), 2.36 (s superimposed, 3H, CH_3_C=N-), 0.78 (t, *J* = 6.6 Hz, 6H, N-(CH_2_CH_3_)_2_). ^13^C-NMR (50 MHz, DMSO-*d_6_*): 178.17, 157.61, 154.61, 148.61, 141.81, 135.75, 131.08, 130.87, 129.33 (2C), 128.31, 120.32, 117.01, 113.56 (2C), 109.31, 54.66, 51.33, 46.42 (2C), 41.03, 31.99, 13.95, 11.31 (2C). Anal. calcd. for C_24_H_32_N_6_OS: % C 63.69, H 7.13, N 18.57, S 7.08; found: % C 63.51, H 6.77, N 18.84, S 7.16.

*2-{1-[2-(4-Chlorobenzyl)-1-[2-(N,N-diethylamino)ethyl]-1H-benzo[d]imidazol-5-yl]-ethylidene}hydrazine-1-carbothioamide (**6**)*: Yield: 37%; m.p. 190–192 °C. ^1^H NMR (200 MHz, DMSO-*d_6_*): 10.18 (s, 1H, NH), 8.25 (s, 1H, NH_2_), 8.11 (s, 1H, H(4)benz.), 7.96 (s superimposed, 1H, NH_2_), 8.10–7.85 (m superimposed, 1H, H(7)benz.), 7.48–7.21 (m, 5H, H(6)benz. and H(2′,3′,5′,6′)arom.), 4.35 (s, 2H, CH_2_-Ar), 4.20 (pseudo s, 2H, CH_2_CH_2_-N(CH_2_CH_3_)_2_), 2.60-2.19 (m superimposed to DMSO, 6H, 2H CH_2_CH_2_-N(CH_2_CH_3_)_2_ and 4H CH_2_CH_2_-N(CH_2_CH_3_)_2_), 2.36 (s superimposed, 3H, CH_3_C=N-), 0.76 (t, *J* = 6.6 Hz, 6H, N(CH_2_CH_3_)_2_). ^13^C-NMR (50 MHz, DMSO-*d_6_*): 178.13, 153.94, 148.59, 141.92, 135.64 (2C), 131.12 (2C), 130.38 (2C), 128.03 (2C), 120.29, 117.09, 109.52, 51.93, 46.43 (2C), 41.08, 32.03, 14.01, 11.31 (2C). Anal. calcd. for C_23_H_29_ClN_6_S: % C 60.44, H 6.40, N 18.39, S 7.01; found: % C 60.57, H 6.45, N 18.52, S 7.10.

*2-(1-{1-[2-(N,N-Diethylamino)ethyl]-2-(4-ethoxybenzyl)-1H-benzo[d]imidazol-5-yl}-ethylidene)hydrazine-1-carbothioamide (**7**)*: Yield: 47%; m.p. 189–192.5 °C. ^1^H NMR (200 MHz, DMSO-*d6)*: 10.18 (s, 1H, NH), 8.24 (s, 1H, NH_2_), 8.10 (s, 1H, H(4)benz.), 7.94 (s superimposed, 1H, NH_2_), 8.05–7.85 (m superimposed, 1H, H(7)benz.), 7.42 (d, *J* = 8.6 Hz, 1H, H(6)benz.), 7.19 (d, *J* = 8.4 Hz, 2H, H(3′,5′)arom.), 6.87 (d, *J* = 8.4 Hz, 2H, H(2′,6′)arom.), 4.27 (s superimposed, 2H, CH_2_-Ar), 4.18 (pseudo s superimposed, 2H, CH_2_CH_2_-N(CH_2_CH_3_)_2_), 3.98 (q, *J* = 7 Hz, 2H, OCH_2_CH_3_), 2.60-2.23 (m superimposed to DMSO, 6H, 2H CH_2_CH_2_-N(CH_2_CH_3_)_2_ and 4H CH_2_CH_2_-N(CH_2_CH_3_)_2_), 2.37 (s superimposed, 3H, CH_3_C=N-), 1.30 (t, *J* = 6.8 Hz, 3H, OCH_2_CH_3_), 0.78 (t, *J* = 6.6 Hz, 6H, N(CH_2_CH_3_)_2_). ^13^C-NMR (50 MHz, DMSO-*d_6_*): 178.20, 156.87, 154.60, 148.60, 141.85, 135.75, 130.89, 129.32 (2C), 128.19, 120.32, 117.03, 114.06 (2C), 109.31, 62.55, 51.46, 46.41 (2C), 41.06, 32.01, 14.25, 13.97, 11.32 (2C). Anal. calcd. for C_25_H_34_N_6_OS: % C 64.35, H 7.34, N 18.01, S 6.87; found: % C 64.30, H 7.51, N 18.10, S 6.77.

*2-(1-{2-Benzyl-1-[3-(N,N-diethylamino)propyl]-1H-benzo[d]imidazol-5-yl}ethylidene)-hydrazine-1-carbothioamide (**8**)*: Yield: 38%; m.p. 168–169 °C. ^1^H NMR (200 MHz, DMSO-*d6)*: 10.19 (s, 1H, NH), 8.27 (s, 1H, NH_2_), 8.14 (s, 1H, H(4)benz.), 7.97 (s superimposed, 1H, NH_2_), 8.03–7.87 (m superimposed, 1H, H(7)benz.), 7.47 (d, *J* = 8.2 Hz, 1H, H(6)benz.), 7.31 (s, 5H, H(2′,3′,4′,5′,6′)arom.), 4.34 (s, 2H, CH_2_-Ar), 4.19 (pseudo s, 2H, CH_2_CH_2_CH_2_-N(CH_2_CH_3_)_2_), 2.37 (s, 3H, CH_3_C=N-), 2.45–2.19 (m, 9H, 4H N(CH_2_CH_3_)_2_, 2H CH_2_CH_2_CH_2_-N(CH_2_CH_3_)_2_ and 3H CH_3_C=N-), 1.63 (pseudo s, 2H, CH_2_CH_2_CH_2_-N(CH_2_CH_3_)_2_), 0.90 (pseudo s, 6H, N(CH_2_CH_3_)_2_). ^13^C-NMR (50 MHz, DMSO-*d_6_*): 178.20, 153.90, 148.58, 141.93, 136.59, 135.60, 131.02, 128.25 (2C), 128.16 (2C), 126.23, 120.45, 117.11, 109.31, 48.66, 45.56 (2C), 41.03, 32.65, 26.16, 14.01, 11.05 (2C). Anal. calcd. for C_24_H_32_N_6_S: % C 66.02, H 7.39, N 19.25, S 7.34; found % C 65.84, H 7.53, N 19.37, S 7.58.

*2-(1-{1-[3-(N,N-Diethylamino)propyl]-2-(4-methoxybenzyl)-1H-benzo[d]imidazol-5-yl}ethylidene)hydrazine-1-carbothioamide (**9**)*: Yield: 38%; m.p. 180.7–182.9 °C. ^1^H NMR (200 MHz, DMSO-*d_6_*): 10.18 (s, 1H, NH), 8.24 (s, 1H, NH_2_), 8.12 (s, 1H, H(4)benz.), 7.95 (s superimposed, 1H, NH_2_), 8.03-7.85 (m superimposed, 1H, H(7)benz.), 7.45 (d, *J* = 8.6 Hz, 1H, H(6)benz.), 7.21 (d, *J* = 8.4 Hz, 2H, H(3′,5′)arom.), 6.88 (d, *J* = 8.2 Hz, 2H, H(2′,6′)arom.), 4.26 (s superimposed, 2H, CH_2_-Ar), 4.16 (pseudo s superimposed, 2H, CH_2_CH_2_CH_2_-N(CH_2_CH_3_)_2_), 3.71 (s, 3H, OCH_3_), 2.37 (s, 3H, CH_3_C=N-), 2.42-2.17 (m, 6H, 4H N(CH_2_CH_3_)_2_ and 2H CH_2_CH_2_CH_2_-N(CH_2_CH_3_)_2_), 1.61 (pseudo s, 2H, CH_2_CH_2_CH_2_-N(CH_2_CH_3_)_2_), 0.89 (t, *J* = 6.8 Hz, 6H, N(CH_2_CH_3_)_2_). ^13^C-NMR (50 MHz, DMSO-*d_6_*): 178.20, 157.62, 154.23, 148.60, 141.92, 135.63, 130.97, 129.23 (2C), 128.34, 120.40, 117.07, 113.55 (2C), 109.26, 54.62, 48.75, 45.56 (2C), 41.04, 31.84, 26.23, 14.00, 11.16 (2C). Anal. calcd. for C_25_H_34_N_6_OS: % C 64.35, H 7.34, N 18.01, S 6.87; found % C 64.19, H 7.74, N 17.96, S 6.85.

*2-(1-{2-[(2H-Benzo[d][1,2,3]triazol-2-yl)methyl]-1-[2-(N,N-dimethylamino)ethyl]-1H-benzo[d]imidazol-5-yl}ethylidene)hydrazine-1-carbothioamide (**18**)*: Yield: 46%; m.p. 214–217 °C. ^1^H NMR (200 MHz, DMSO-*d_6_*): 10.19 (s, 1H, NH), 8.26 (s, 1H, NH_2_), 8.14 (s, 1H, H(4)benz.), 8.07–7.85 (m, 4H, 1H H(7)benz., 1H NH_2_ and 2H H(4′,7′)bzt.), 7.55 (d, *J* = 8.6 Hz, 1H, H(6)benz.), 7.52–7.39 (m, 2H, H(5′,6′)bzt.), 6.44 (s, 2H, CH_2_-Ar), 4.44 (pseudo s, 2H, CH_2_CH_2_-N(CH_3_)_2_), 2.43-2.29 (m superimposed, 2H, CH_2_CH_2_-N(CH_3_)_2_), 2.36 (s, 3H, CH_3_C=N-), 2.14 (s, 6H, N(CH_3_)_2_). ^13^C-NMR (50 MHz, DMSO-*d_6_*): 178.24, 148.60, 148.23, 143.58 (2C), 141.49, 135.68, 131.65, 126.43 (2C), 121.45, 117.74, 117.54 (2C), 109.91, 57.79, 52.31, 45.08 (2C), 41.84, 13.96. Anal. calcd. for C_21_H_25_N_9_S: % C 57.91, H 5.79, N 28.94, S 7.36; found % C 57.90, H 5.90, N 28.64, S 7.22.

*2-(1-{2-[(2H-Benzo[d][1,2,3]triazol-2-yl)methyl]-1-[3-(N,N-dimethylamino)propyl]-1H-benzo[d]imidazol-5-yl}ethylidene)hydrazine-1-carbothioamide (**19**)*: Yield: 31%; m.p. 212–214 °C. ^1^H NMR (200 MHz, DMSO-*d_6_*): 10.19 (s, 1H, NH), 8.26 (s, 1H, -NH_2_), 8.14 (s, 1H, H(4)benz.), 8.06–7.80 (m, 4H, 1H H(7)benz., 1H NH_2_ and 2H H(4′,7′)bzt.), 7.68–7.50 (m, 1H, H(6)benz.), 7.47 (pseudo s superimposed, 2H, H(5′,6′)bzt.), 6.47 (s, 2H, CH_2_-Ar), 4.39 (pseudo s, 2H, CH_2_CH_2_CH_2_-N(CH_3_)_2_), 2.36 (s, 3H, CH_3_C=N-), 2.21–1.90 (m superimposed, 2H, CH_2_CH_2_CH_2_-N(CH_3_)_2_), 2.05 (s superimposed, 6H, N(CH_3_)_2_), 1.73 (pseudo s, 2H, CH_2_CH_2_CH_2_-N(CH_3_)_2_). ^13^C-NMR (50 MHz, DMSO-*d_6_*): 178.25, 148.38, 148.25, 143.56 (2C), 141.61, 135.48, 131.66, 126.40 (2C), 121.47, 117.77, 117.53 (2C), 109.94, 54.86, 52.01, 44.41 (2C), 41.11, 26.30, 13.96. Anal. calcd. for C_22_H_27_N_9_S: % C 58.77, H 6.05, N 28.04, S 7.13; found % C 58.88, H 5.96, N 27.78, S 7.33.

*2-(1-{2-[(2H-Benzo[d][1,2,3]triazol-2-yl)methyl]-1-[3-(N,N-diethylamino)propyl]-1H-benzo[d]imidazol-5-yl}ethylidene)hydrazine-1-carbothioamide (**21**)*: Yield: 78%; m.p. 214–216 °C. ^1^H NMR (200 MHz, DMSO-*d_6_*): 10.18 (s, 1H, NH), 8.27 (s, 1H, NH_2_), 8.13 (s, 1H, H(4)benz.), 8.09–7.86 (m, 4H, 1H H(7)benz., 1H NH_2_ and 2H H(4′,7′)bzt.), 7.55 (d, *J* = 8.8 Hz, 1H, H(6)benz.), 7.53–7.39 (m, 2H, H(5′,6′)bzt.), 6.47 (s, 2H, CH_2_-Ar), 4.40 (pseudo s, 2H, CH_2_CH_2_-N(CH_2_CH_3_)_2_), 2.67–2.25 (m superimposed to DMSO, 6H, 2H CH_2_CH_2_-N(CH_2_CH_3_)_2_ and 4H N(CH_2_CH_3_)_2_), 2.36 (s, 3H, CH_3_C=N-), 0.77 (t, *J* = 6.8 Hz, 6H, N(CH_2_CH_3_)_2_). ^13^C-NMR (50 MHz, DMSO-*d_6_*): 178.25, 148.82, 148.25, 143.59 (2C), 141.52, 135.58, 131.54, 126.40 (2C), 121.34, 117.74, 117.54 (2C), 109.88, 52.43, 51.68, 46.55 (2C), 42.29, 13.91, 11.26 (2C). Anal. calcd. for C_23_H_29_N_9_S: % C 59.59, H 6.31, N 27.19, S 6.92; found % C 59.61, H 6.54, N 27.06, S 7.15.

*2-(1-{2-[(2H-Benzo[d][1,2,3]triazol-1-yl)methyl]-1-[3-(N,N-dimethylamino)propyl]-1H-benzo[d]imidazol-5-yl}ethylidene)hydrazine-1-carbothioamide (**22**)*: Yield: 34%; m.p. 201–203 °C. ^1^H NMR (200 MHz, DMSO-*d_6_*): 10.17 (s, 1H, NH), 8.23 (s, 1H, NH_2_), 8.12 (s superimposed, 1H, H(4)benz.), 8.18–8.05 (m, 1H, H(7′)bzt.), (s, 1H, NH_2_), 8.02–7.93 (m, 2H, 1H H(4′)bzt. and 1H NH_2_), 7.89 (d, *J* = 8.6 Hz, 1H H(7)benz.), 7.63-7.50 (m, 2H, H(5′,6′)bzt.), 7.45 (d, *J* = 8.4 Hz, 1H, H(6)benz.), 6.45 (s, 2H, CH_2_-Ar), 4.42 (pseudo s, 2H, CH_2_CH_2_CH_2_-N(CH_3_)_2_), 2.33 (s, 3H, CH_3_C=N-), 2.35–1.90 (m superimposed, 2H, CH_2_CH_2_CH_2_-N(CH_3_)_2_), 2.13 (s, 6H, N(CH_3_)_2_), 1.79 (pseudo s, 2H, CH_2_CH_2_CH_2_-N(CH_3_)_2_). ^13^C-NMR (50 MHz, DMSO-*d_6_*): 178.21, 148.98, 148.25, 144.91, 141.50, 135.61, 132.87, 131.58, 127.23, 123.80, 121.39, 118.85, 117.62, 110.61, 109.89, 54.86, 44.43 (2C), 44.03, 41.02, 26.27, 13.90. Anal. calcd. for C_22_H_27_N_9_S: % C 58.77, H 6.05, N 28.04, S 7.13; found % C 58.92, H 6.18, N 28.06, S 6.84.

#### 3.1.3. General Procedure for the Preparation of Semicarbazones

To a solution of the proper 5-acetyl benzimidazole (0.80 mmol) in ethanol (2 mL), a solution of semicarbazide hydrochloride (2.4 mmol) previously dissolved in 8 mL of a 1N solution of sodium acetate was added. The mixture was refluxed for 4h under stirring. After evaporation of the solvent, the oily residue was treated with warm water to get rid of the remaining semicarbazide. The crude was purified by CC (SiO_2_, CH_2_Cl_2_+5% DEA), obtaining the title compound as a white solid.

2-(1-{1-[2-(N,N-Dimethylamino)ethyl]-2-(4-methoxybenzyl)-1H-benzo[d]imidazol-5-yl}-ethylidene)hydrazine-1-carboxamide (**10**): Yield: 69%; m.p. 203–205 °C. ^1^H NMR (200 MHz, DMSO-*d_6_*): 9.28 (s, 1H, NH), 7.98 (s, 1H, H(4)benz.), 7.83 (d, *J* = 8.8 Hz, 1H, H(7)benz.), 7.43 (d, *J* = 8.6 Hz, 1H, H(6)benz.), 7.21 (d, *J* = 8.6 Hz, 2H, H(3′,5′)arom.), 6.89 (d, *J* = 8.6 Hz, 2H, H(2′,6′)arom.), 6.50 (broad s, 2H, NH_2_), 4.24 (s superimposed, 2H, CH_2_-Ar), 4.39–4.17 (m, 2H, CH_2_CH_2_-N(CH_3_)_2_), 3.72 (s, 3H, OCH_3_), 2.41-2.20 (m superimposed, 2H, CH_2_CH_2_-N(CH_3_)_2_), 2.25 (s superimposed, 3H, CH_3_C=N-), 2.12 (s superimposed, 6H, N(CH_3_)_2_). ^13^C-NMR (50 MHz, DMSO-*d_6_*): 157.63, 157.09, 154.20, 144.64, 141.86, 135.23, 131.78, 129.37 (2C), 128.34, 119.88, 116.18, 113.56 (2C), 109.25, 57.45, 54.67, 45.04 (2C), 41.12, 31.91, 13.38. Anal. calcd. for C_22_H_28_N_6_O_2_: % C 64.68, H 6.91, N 20.57; found % C 64.70, H 6.67, N 20.35.

*2-{1-[2-(4-Chlorobenzyl)-1-[2-(N,N-dimethylamino)ethyl]-1H-benzo[d]imidazol-5-yl}-ethylidene)hydrazine-1-carboxamide (**11**)*: Yield: 59%; m.p. 217–220 °C. ^1^H NMR (200 MHz, DMSO-*d_6_*): 9.29 (s, 1H, NH), 7.99 (s, 1H, H(4)benz.), 7.83 (d, *J* = 8.2 Hz, 1H, H(7)benz.), 7.48–7.24 (m, 5H, H(7)benz. and H(2′,3′,5′,6′)arom.), 6.52 (broad s, 2H, NH_2_), 4.33 (s superimposed, 2H, CH_2_-Ar), 4.41-4.17 (m superimposed, 2H, CH_2_CH_2_-N(CH_3_)_2_), 3.72 (s, 3H, OCH_3_), 2.42-2.30 (m, 2H, CH_2_CH_2_-N(CH_3_)_2_), 2.26 (s, 3H, CH_3_C=N-), 2.13 (s, 6H, N(CH_3_)_2_). ^13^C-NMR (50 MHz, DMSO-*d_6_*): 157.11, 153.57, 144.64, 141.83, 135.66, 135.16, 131.88, 130.87, 130.39 (2C), 128.03 (2C), 120.01, 116.23, 109.34, 57.57, 45.05 (2C), 41.18, 31.92, 13.37. Anal. calcd. for C_21_H_25_ClN_6_O: % C 61.08, H 6.10, N 20.35; found % C 61.08, H 5.95, N 20.65.

*2-(1-{1-[2-(N,N-Dimethylamino)ethyl]-2-(4-ethoxybenzyl)-1H-benzo[d]imidazol-5-yl}-ethylidene)hydrazine-1-carboxamide (**12**)*: Yield: 38%; m.p. 210–213 °C. ^1^H NMR (200 MHz, DMSO-*d_6_*): 9.28 (s, 1H, NH), 7.99 (s, 1H, H(4)benz.), 7.83 (d, *J* = 8.6 Hz, 1H, H(7)benz.), 7.43 (d, *J* = 8.6 Hz, 1H, H(6)benz.), 7.19 (d, *J* = 7.4 Hz, 2H, H(3′,5′)arom.), 6.87 (d, *J* = 7.6 Hz, 2H, H(2′,6′)arom.), 6.50 (broad s, 2H, NH_2_), 4.24 (pseudo s, 4H, CH_2_-Ar and CH_2_CH_2_-N(CH_3_)_2_), 3.98 (q, *J* = 6.8 Hz, 2H, OCH_2_CH_3_), 2.41-2.18 (m superimposed, 2H, CH_2_CH_2_-N(CH_3_)_2_), 2.25 (s superimposed, 3H, CH_3_C=N-), 2.12 (s, 6H, N(CH_3_)_2_), 1.30 (t, *J* = 6.8 Hz, 3H, OCH_2_CH_3_). ^13^C-NMR (50 MHz, DMSO-*d_6_*): 157.09, 156.87, 154.20, 144.64, 141.87, 135.23, 131.79, 129.34 (2C), 128.21, 119.88, 116.19, 114.06 (2C), 109.25, 62.56, 57.44, 45.03 (2C), 41.13, 31.92, 14.25, 13.38. Anal. calcd. for C_23_H_30_N_6_O_2_: % C 65.38, H 7.16, N 19.89; found % C 65.56, H 7.48, N 19.70.

*2-{1-[2-(4-Chlorobenzyl)-1-[3-(N,N-dimethylamino)propyl]-1H-benzo[d]imidazol-5-yl}-ethylidene)hydrazine-1-carboxamide (**13**)*: Yield: 67%; m.p. 211–212.5 °C. ^1^H NMR (200 MHz, DMSO-*d_6_*): 9.29 (s, 1H, NH), 8.00 (s, 1H, H(4)benz.), 7.83 (d, *J* = 8.2 Hz, 1H, H(7)benz.), 7.47–7.20 (m, 5H, H(6)benz. and H(2′,3′,5′,6′)arom.), 6.51 (broad s, 2H, NH_2_), 4.34 (s, 2H, CH_2_-Ar), 4.26–4.13 (m, 2H, CH_2_CH_2_CH_2_-N(CH_3_)_2_), 2.26 (s, 3H, CH_3_C=N-), 2.20–1.98 (m, 8H, 2H CH_2_CH_2_CH_2_-N(CH_3_)_2_ and 6H N(CH_3_)_2_), 1.81–1.58 (m, 2H, CH_2_CH_2_CH_2_-N(CH_3_)_2_. ^13^C-NMR (50 MHz, DMSO-*d_6_*): 157.08, 153.40, 144.58, 141.90, 135.69, 135.07, 131.87, 130.89, 130.29 (2C), 128.06 (2C), 119.98, 116.27, 109.28, 55.17, 44.58 (2C), 40.71, 31.80, 26.59, 13.37. Anal. calcd. for C_22_H_27_ClN_6_O: % C 61.89, H 6.37, N 19.68; found % C 62.09, H 6.63, N 19.89.

*2-(1-{1-[2-(N,N-Diethylamino)ethyl]-2-(4-methoxybenzyl)-1H-benzo[d]imidazol-5-yl}-ethylidene)hydrazine-1-carboxamide (**14**)*: Yield: 72%; m.p. 207–209 °C. ^1^H NMR (200 MHz, DMSO-*d_6_*): 9.26 (s, 1H, NH), 7.98 (s, 1H, H(4)benz.), 7.84 (d, *J* = 8.6 Hz, 1H, H(7)benz.), 7.41 (d, *J* = 8.6 Hz, 1H, H(6)benz.), 7.20 (d, *J* = 8.8 Hz, 2H, H(3′,5′)arom.), 6.89 (d, *J* = 8.8 Hz, 2H, H(2′,6′)arom.), 6.49 (broad s, 2H, NH_2_), 4.27 (s, 2H, CH_2_-Ar), 4.22–4.09 (m, 2H, CH_2_CH_2_-N(CH_2_CH_3_)_2_), 3.72 (s, 3H, OCH_3_), 2.50–2.36 (m superimposed to DMSO, 6H, 2H CH_2_CH_2_-N(CH_2_CH_3_)_2_ and 4H CH_2_CH_2_-N(CH_2_CH_3_)_2_), 2.26 (s, 3H, CH_3_C=N-), 0.78 (t, *J* = 7.0 Hz, 6H, N(CH_2_CH_3_)_2_). ^13^C-NMR (50 MHz, DMSO-*d_6_*): 157.63, 157.09, 154.36, 144.67, 141.88, 135.20, 131.68, 129.30 (2C), 128.40, 119.76, 116.19, 113.57 (2C), 109.21, 54.66, 51.39, 46.45 (2C), 41.13, 32.02, 13.34, 11.39 (2C). Anal. calcd. for C_24_H_32_N_6_O_2_: % C 66.03, H 7.39, N 19.25; found % C 65.98, H 7.42, N 19.56.

*2-{1-[2-(4-Chlorobenzyl)-1-[2-(N,N-diethylamino)ethyl]-1H-benzo[d]imidazol-5-yl}-ethylidene)hydrazine-1-carboxamide (**15**)*: Yield: 56%; m.p. 196.5–198.5 °C. ^1^H NMR (200 MHz, DMSO-*d6)*: 9.27 (s, 1H, NH), 7.97 (s, 1H, H(4)benz.), 7.85 (d, *J* = 8.6 Hz, 1H, H(7)benz.), 7.51–7.24 (m, 5H, H(6)benz. and H(2′,3′,5′,6′)arom.), 6.50 (broad s, 1H, NH_2_), 4.34 (s, 2H, CH_2_-Ar), 4.19 (pseudo s, 2H, CH_2_CH_2_-N(CH_2_CH_3_)_2_), 2.58-2.33 (m superimposed to DMSO, 6H, 2H CH_2_CH_2_-N(CH_2_CH_3_)_2_ and 4H CH_2_CH_2_-N(CH_2_CH_3_)_2_), 2.25 (s superimposed, 3H, CH_3_C=N-), 0.76 (t, *J* = 6.4 Hz, 6H, N(CH_2_CH_3_)_2_). ^13^C-NMR (50 MHz, DMSO-*d_6_*): 157.09, 153.73, 144.62, 141.81, 135.68, 135.12, 131.74, 130.87, 130.34 (2C), 128.03 (2C), 119.88, 116.23, 109.32, 51.41, 46.43 (2C), 42.10, 32.04, 13.32, 11.23 (2C). Anal. calcd. for C_23_H_29_ClN_6_O: % C 62.65, H 6.63, N 19.06; found: % C 62.58, H 6.50, N 19.26.

*2-(1-{1-[3-(N,N-Diethylamino)propyl]-2-benzyl-1H-benzo[d]imidazol-5-yl}ethylidene)-hydrazine-1-carboxamide (**16**)*: Yield: 32%; m.p. 185–189 °C. ^1^H NMR (200 MHz, DMSO-*d6)*: 9.28 (s, 1H, NH), 8.01 (s, 1H, H(4)benz.), 7.82 (d, *J* = 8.6 Hz, 1H, H(7)benz.), 7.45 (d, *J* = 8.6 Hz, 1H, H(6)benz.), 7.30 (pseudo s, 5H, H(2′,3′,5′,6′)arom.), 6.50 (broad s, 1H, NH_2_), 4.33 (s, 2H, CH_2_-Ar), 4.17 (pseudo s, 2H, CH_2_CH_2_CH_2_-N(CH_2_CH_3_)_2_), 2.47–2.27 (m superimposed, 6H, 2H CH_2_CH_2_CH_2_-N(CH_2_CH_3_)_2_ and 4H CH_2_CH_2_-N(CH_2_CH_3_)_2_), 2.26 (s superimposed, 3H, CH_3_C=N-), 1.62 (pseudo s, 2H, CH_2_CH_2_CH_2_-N(CH_2_CH_3_)_2_), 0.90 (pseudo s, 6H, N(CH_2_CH_3_)_2_). ^13^C-NMR (50 MHz, DMSO-*d_6_*): 157.07, 153.64, 144.62, 141.95, 136.65, 135.04, 131.80, 128.22 (2C), 128.15 (2C), 126.20, 119.89, 116.26, 109.25, 48.69, 45.56 (2C), 41.10, 32.65, 26.17, 13.36, 11.06 (2C). Anal. calcd. for C_24_H_32_N_6_O: % C 68.54, H 7.67, N 19.98; found: % C 68.35, H 7.84, N 20.30.

*2-(1-{1-[3-(N,N-Diethylamino)propyl]-2-(4-methoxybenzyl)-1H-benzo[d]imidazol-5-yl}-ethylidene)hydrazine-1-carboxamide (**17**)*: Yield: 32%; m.p. 190–192 °C. ^1^H NMR (200 MHz, DMSO-*d_6_*): 9.28 (s, 1H, NH), 7.99 (s, 1H, H(4)benz.), 7.83 (d, *J* = 8.6 Hz, 1H, H(7)benz.), 7.44 (d, *J* = 8.6 Hz, 1H, H(6)benz.), 7.21 (d, *J* = 7.0 Hz, 2H, H(3′,5′)arom.), 6.88 (d, *J* = 7.2 Hz, 2H, H(2′,6′)arom.), 6.51 (broad s, 2H, NH_2_), 4.25 (s, 2H, CH_2_-Ar), 4.22-4.07 (m superimposed, 2H, CH_2_CH_2_CH_2_-N(CH_2_CH_3_)_2_), 3.71 (s, 3H, OCH_3_), 2.46–2.20 (m superimposed, 6H, 2H CH_2_CH_2_CH_2_-N(CH_2_CH_3_)_2_ and 4H N(CH_2_CH_3_)_2_), 2.26 (s, 3H, CH_3_C=N-), 1.60 (pseudo s, 2H, CH_2_CH_2_CH_2_-N(CH_2_CH_3_)_2_), 0.89 (pseudo s, 6H, N(CH_2_CH_3_)_2_). ^13^C-NMR (50 MHz, DMSO-*d_6_*): 157.61, 157.07, 153.98, 144.62, 141.95, 135.07, 131.75, 129.22 (2C), 128.40, 119.84, 116.23, 113.55 (2C), 109.20, 54.61, 48.76, 45.56 (2C), 41.29, 31.85, 26.24, 13.37, 11.15 (2C). Anal. calcd. for C_25_H_34_N_6_O_2_: % C 66.64, H 7.61, N 18.65; found % C 66.38, H 7.56, N 18.31.

*2-(1-{2-[(2H-Benzo[d][1,2,3]triazol-2-yl)methyl]-1-[2-(N,N-dimethylamino)ethyl]-1H-benzo[d]imidazol-5-yl}ethylidene)hydrazine-1-carboxamide (**20**)*: Yield: 48%; m.p. 209–212 °C. ^1^H NMR (200 MHz, DMSO-*d_6_*): 9.31 (s, 1H, NH), 8.03 (s, 1H, H(4)benz.), 8.00–7.85 (m, 3H, 1H H(7)benz., and 2H H(4′,7′)bzt.), 7.59–7.41 (m, 3H, 1H H(6)benz. and 2H H(5′,6′)bzt.), 6.53 (broad s, 2H, NH_2_), 6.43 (s, 2H, CH_2_-Ar), 4.53-4.34 (m, 2H, CH_2_CH_2_-N(CH_3_)_2_), 2.46-2.34 (m, 2H, CH_2_CH_2_-N(CH_3_)_2_), 2.24 (s, 3H, CH_3_C=N-), 2.14 (s, 6H, N(CH_3_)_2_). ^13^C-NMR (50 MHz, DMSO-*d_6_*): 157.03, 148.32, 144.29, 143.57 (2C), 141.52, 135.14, 132.42, 126.42 (2C), 120.95, 117.74, 117.54 (2C), 116.85, 109.85, 57.77, 52.30, 45.08 (2C), 41.56, 13.31. Anal. calcd. for C_21_H_25_N_9_O: % C 60.13, H 6.01, N 30.05; found % C 60.39, H 6.35, N 30.80.

#### 3.1.4. General Procedure for the Preparation of Hydrazones

A solution of NH_2_NH_2_ · H_2_O (2.5 mmol) in 3 mL of water was refluxed for 5 h with a solution of the proper 5-acetyl benzimidazole (0.50 mmol) in 2.5 mL of ethanol with stirring. At room temperature, 5 mL of water were added and the solution was kept at 0–5 °C overnight. The expected product was directly separated from the solution as an amorphous solid that was filtered and crystallized as a white solid from anhydrous Et_2_O.

*1-[2-(N,N-Dimethylamino)ethyl]-2-(4-methoxybenzyl)-5-(1-hydrazineylideneethyl)-1H-benzo[d]imidazole (**23**)*: Yield: 53%; m.p. 175.5–176.5 °C. ^1^H NMR (200 MHz, DMSO-*d6)*: 8.11 (s, 1H, H(4)benz.), 7.92 (pseudo s, 1H, H(7)benz.), 7.55 (pseudo s, 1H, H(6)benz.), 7.22 (pseudo s, 2H, H(3′,5′)arom.), 6.91 (pseudo s, 2H, H(2′,6′)arom.), 6.27 (broad s, 2H, NH_2_), 4.39–4.10 (m, 4H, CH_2_-Ar and CH_2_CH_2_-N(CH_3_)_2_), 3.73 (s, 3H, OCH_3_), 2.61-2.28 (m superimposed to DMSO, 5H, 2H CH_2_CH_2_-N(CH_3_)_2_ and 3H CH_3_C=N-), 2.14 (s, 6H, N(CH_3_)_2_). ^13^C-NMR (50 MHz, DMSO-*d_6_*): 158.17, 157.66, 154.57, 141.84, 136.09, 131.58, 129.36 (2C), 128.28, 120.17, 116.89, 113.60 (2C), 109.53, 57.42, 54.67, 45.05 (2C), 41.13, 31.93, 14.53. Anal. calcd. for C_21_H_27_N_5_O: % C 69.01, H 7.45, N 19.16; found % C 69.20, H 7.35, N 19.16.

*1-[2-(N,N-diethylamino)ethyl]-2-(4-ethoxybenzyl)-5-(1-hydrazineylideneethyl)-1H-benzo[d]imidazole (**24**)*: Yield: 44%; m.p. 80–82 °C. ^1^H NMR (200 MHz, DMSO-*d6)*: 8.11 (s, 1H, H(4)benz.), 7.87 (d, *J* = 8.6 Hz, 1H, H(7)benz.), 7.47 (d, *J* = 8.6 Hz, 1H, H(6)benz.), 7.21 (pseudo s, 2H, H(3′,5′)arom.), 6.89 (pseudo s, 2H, H(2′,6′)arom.), 6.32 (broad s, 2H, NH_2_), 4.41–4.13 (m, 4H, 2H CH_2_-Ar and 2H CH_2_CH_2_-N(CH_2_CH_3_)_2_), 3.97 (pseudo s, 2H, OCH_2_CH_3_), 2.58–2.27 (m superimposed to DMSO, 6H, 2H CH_2_CH_2_-N(CH_2_CH_3_)_2_ and 4H N(CH_2_CH_3_)_2_), 2.29 (s superimposed, 3H, CH_3_C=N-), 1.31 (t, *J* = 6.8 Hz, 3H, OCH_2_CH_3_), 0.78 (t, *J* = 6.8 Hz, 6H, N(CH_2_CH_3_)_2_). ^13^C-NMR (50 MHz, DMSO-*d_6_*): 158.18, 157.64, 154.24, 141.87, 135.89, 131.67, 129.23 (2C), 128.34, 120.04, 116.89, 113.78 (2C), 109.57, 62.67, 57.43, 45.09 (2C), 41.12, 31.91, 14.44, 13.98, 11.23 (2C). Anal. calcd. for C_24_H_33_N_5_O: % C 70.73, H 8.16, N 17.18; found % C 70.67, H 8.47, N 17.43.

*2-[(Benzotriazol-2-yl)methyl]-1-[2-(N,N-diethylamino)ethyl]-5-(1-hydrazineylideneethyl)-1H-benzo[d]imidazole (**25**)*: Yield: 35%; m.p. 128–130 °C. ^1^H NMR (200 MHz, DMSO-*d6)*: 8.01–7.87 (m, 2H, H(4′,7′)bzt.), 7.76 (s, 1H, H(4)benz.), 7.70 (d, *J* = 8.8 Hz, 1H, H(7)benz.), 7.60–7.38 (m, 3H, 1H H(6)benz. and 2H H(5′,6′)bzt.), 6.43 (s, 2H, CH_2_-Ar), 6.26 (broad s, 2H, NH_2_), 4.43-4.21 (m, 2H, CH_2_CH_2_-N(CH_2_CH_3_)_2_), 2.64–2.48 (m superimposed to DMSO, 2H, CH_2_CH_2_-N(CH_2_CH_3_)_2_), 2.42 (q, *J* = 6.8 Hz, 4H, N(CH_2_CH_3_)_2_), 2.08 (s, 3H, CH_3_C=N-), 1.31 (t, *J* = 6.8 Hz, 3H, OCH_2_CH_3_), 0.76 (t, *J* = 6.8 Hz, 6H, N(CH_2_CH_3_)_2_). ^13^C-NMR (50 MHz, DMSO-*d_6_*): 158.04, 148.86, 147.97, 143.57, 141.42, 135.84, 134.07, 132.11, 126.49 (2C), 121.15, 117.51 (2C), 110.16, 52.31, 51.61, 46.49 (2C), 42.59, 14.52, 11.15 (2C). Anal. calcd. for C_22_H_28_N_8_: % C 65.32, H 6.98, N 27.70; found % C 65.57, H 6.69, N 27.58.

### 3.2. Biology

#### Antiviral Assays

The detailed antiviral procedures can be found elsewhere [24,25,26,27]. Briefly, human influenza viruses A/HK/7/87 (A/H3N2), A/Ned/378/05 (A/H1N1) and B/Ned/537/05 (all from R. Fouchier, Rotterdam, the Netherlands) were tested in Madin–Darby canine kidney (MDCK) cells, a kind gift from M. Matrosovich (Marburg, Germany). Respiratory syncytial virus (RSV; strain Long) was evaluated on human epithelial type 2 cells (Hep-2), and human coronavirus 229E was assessed on human embryonic lung (HEL) fibroblast cells (all from ATCC). For the other viruses, we refer to our previous report [25]. Semiconfluent cultures of MDCK, Hep-2, Vero or HEL cells in 96-well plates were infected with viruses at a multiplicity of infection of 100 CCID_50_ (50% cell culture infective dose). At the same time, serial compound dilutions were added. The plates were incubated (at 35 °C for influenza and coronavirus, and at 37 °C for the other viruses) for 4 to 6 days, until full-blown cytopathic effect (CPE) was visible. At that time, microscopy was performed to score the viral CPE and compound cytotoxicity (assessed in mock-infected plates). Next, the MTS cell viability reagent (CellTiter 96^®^ AQ_ueous_ One Solution Cell Proliferation Assay from Promega) was added, and after 3 h of incubation at 37 °C, the OD_490nm_ values were measured in a plate reader. The compounds’ EC_50_ (50% antivirally effective concentration) values were calculated by interpolation using semi-log dose response curves. For the MTS data, the percentage of protection against virus was defined as: ((OD_Cpd_)virus − (OD_Contr_)virus))/((OD_Contr_)mock − (OD_Contr_)virus) × 100, where (OD_Cpd_)virus is the OD for a given concentration of the compound in virus-infected cells; (OD_Contr_)virus is the OD for the untreated virus control; and (OD_Contr_)mock is the OD for the untreated mock-infected control. The values for CC_50_ (50% cytotoxic concentration) were also calculated by interpolation using semi-log dose response curves. The percentage of cytotoxicity was defined as: (1 − (OD_Cpd_)mock/((OD_Contr_)mock) × 100, where (OD_Cpd_)mock is the OD for a given concentration of the compound in mock-infected wells.

### 3.3. Molecular Modelling Studies

#### 3.3.1. Docking Calculations

All the compounds were built, parameterized (Gasteiger-Huckel method) and energy minimized within MOE using MMFF94 forcefield [34]. All ligands were used in their protonated state. Docking calculations within the X-ray structure of RSV F protein (pdb code = 5KWW) were done using the LeadIT 2.1.8 software suite (www.biosolveit.com) including the FlexX scoring algorithm, which is based on binding free energy calculations by means of Gibbs–Helmholtz equation [35,36,37]. The software detects the binding site defining a radius of 10 Å far from the co-crystallized ligand, in order to set up a spherical search space for the docking approach. The standard settings for the docking strategy were followed, choosing the so-called hybrid approach (enthalpy and entropy criteria); the related scoring function evaluation is described in the literature [38]. The derived docking poses were prioritized by the score values of the lowest energy pose of the compounds docked to the protein structure. All ligands were refined and rescored by assessment with the algorithm HYDE, included in the LeadIT 2.1.8 software. The HYDE module considers dehydration enthalpy and hydrogen bonding [39,40]. Finally, the reliability of the selected docking poses was assessed using a short ~1 ps run of molecular dynamics (MD) at constant temperature, followed by an all-atom energy minimization (LowModeMD implemented in MOE software). This represents a conformational search method that uses implicit vibrational analysis to focus a MD trajectory along the low-mode vibrations [41,42,43]. This has the effect of searching for minima along the valleys and troughs on the potential energy surface, thereby performing an exhaustive conformational analysis of the ligand–receptor binding site complex, as we previously discussed about other case studies [44,45,46].

#### 3.3.2. In Silico Evaluation of Pharmacokinetic Properties

ADME properties have been predicted by means of Advanced Chemistry Development (ACD) Percepta platform (www.acdlabs.com) named ACD/Labs Percepta software (version 2.0). All of the calculated parameters were derived and evaluated by Percepta on the basis of training libraries, implemented in the software, which include a consistent number of molecules, whose pharmacokinetic and toxicity profiles are known.

## 4. Conclusions

In summary, this study reports the synthesis of a series of (thio)semicarbazone- and hydrazone-containing benzimidazoles for the development of novel antiviral agents which have shown the ability to inhibit the replication of three human respiratory viruses. Acute viral respiratory illnesses are usually the result of infections with a heterogeneous group of respiratory viruses, including some of the most notable RNA viruses, such as influenza virus, coronavirus and RSV. The relative infections continue to cause frequent morbidity, and sometimes cause severe outcomes, including about 3.9 million deaths worldwide each year, particularly among children under five years, the elderly and immunocompromised individuals [47,48]. This scenario enlightens the serious and important need for identifying new scaffolds that are useful in discovering innovative, highly potent and safe antiviral agents [7].

Interestingly, our antiviral data suggest compounds **6**, **8**, **16** and **17** work as dual virus inhibitors of influenza and coronavirus strains. In these series the (thio)semicarbazone and hydrazone moieties have proven to mediate the observed antiviral activity of 2-benzylbenzimidazoles and 2-[(benzotriazol-1/2-yl)methyl]benzimidazole scaffolds, since the respective chemical precursor 5-acetylbenzimidazoles were found to be less effective or ineffective antiviral agents. It is worth noting that, although the efficacy against human coronavirus (229E) is moderate, these compounds are the first benzimidazole derivatives to be found as active against this virus. Their chemical optimization might acquire greater importance in light of the current outbreak of the novel coronavirus (2019-nCoV); WHO is calling for the urgent setting up of a complex network of strategies, which also look for accelerating the development of diagnostics, vaccines and therapeutics to contain the pandemic proportion of 2019-nCoV infections [49].

Moreover, compounds **25** and **22** proved to be the most potent and safe antivirals among these series, being able to inhibit RSV replication with the same degree of potency of ribavirin, w is the only drug available to treat RSV infections, but its limited efficacy and low margin of safety restrict its use to children at high risk [50]. Docking studies also performed on the best performing RSV F protein inhibitors reported in the literature supported the SAR observed in these series of compounds, and enlightened the efficient binding modes of **25** and **22** at the exposed surface of the RSV F protein, establishing π-π stacking and cation-π interactions with the hydrophobic pocket formed by F137, F140 and F488 residues.

Therefore, the above adequately substituted (thio)semicarbazone- and hydrazone-based benzimidazoles, inhibiting the replication of the aforementioned viruses, may be considered as promising new hits, worthy of further structural optimization for an improved antiviral profile, as a result of the chemical variation of the benzimidazole core: (a) by exploring different side chains in position 1 and/or (b) replacing the benzyl or (benzotriazolyl)methyl moieties with different aromatic or heteroaromatic rings. In particular, with the aim of obtaining more effective and drug-like anti-RSV agents, the design process will be driven by the previously built CoMFA and CoMSIA models, filtering tools predicting the safety trend of any new analogue prior to synthesis [19]. Meanwhile, as some compounds are able to target both influenza virus and coronavirus, their underlying possible common mechanisms of viral inhibition are worth investigation.

## Figures and Tables

**Table 1:** Activities of compounds **6**, **8**, **16**, **17**, **22**, **24** and **25** against three different human respiratory viruses.

Compound	Antiviral EC_50_^a^ (µM)			
	RSV	Influenza A/H1N1	Influenza A/H3N2	Coronavirus 229E
**6**	>100	25	81	38
**8**	>100	38	>100	56
**16**	>100	47	>100	39
**17**	>100	47	>100	41
**22** ^b^	7.0	41	>100	>100
**24**	>100	>100	>100	43
**25**	2.4	>100	>100	>100
Zanamivir	-	0.6	31	-
Ribavirin	6.7	7.4	7.5	-
DS-10,000 ^c^	0.01	-	-	-
UDA ^c^	-	-	-	2.2

^a^ EC_50_: 50% effective concentration or concentration giving 50% protection against virus-induced reduction in cell viability, as determined by the colorimetric formazan-based MTS assay. ^b^ This compound was also active against Coxsackie B4 virus with an EC_50_ value of 20 µM. ^c^ For dextran sulfate MW 10,000 (DS-10,000) and UDA lectin, concentrations are in µg/mL.

**Table 2:** Cytotoxicity of compounds **6**, **8**, **16**, **17**, **22**, **24** and **25** in four mammalian cell lines.

Compound	CC_50_^a^ (µM)			
	Hep-2	HEL	Vero	MDCK
**6**	>100	>100	>100	>100
**8**	>100	>100	44	>100
**16**	>100	>100	>100	>100
**17**	48	>100	43	>100
**22**	>100	100	>100	>100
**24**	>100	>100	51	35
**25**	>100	>100	>100	33
Zanamivir	-	-	-	>100
Ribavirin	>250	-	>250	>100
DS-10,000 ^b^	>100	-	>100	-
UDA ^b^	-	>100	-	-

^a^ CC_50_: 50% cytotoxic concentration based on the MTS cell viability assay. Hep-2: human epithelial type 2 cells; HEL: human embryonic lung fibroblast cells; Vero: African green monkey kidney cells; MDCK: Madin–Darby canine kidney cells. ^b^ For dextran sulfate MW 10,000 (DS-10,000) and UDA lectin, concentrations are in µg/mL.

**Table 3:** Binding affinity values obtained by molecular docking studies of the compounds **JNJ-53718678**, **4**, **19**, **21**, **22**, **24** and **25**.

Protein-Ligand Complex (LeadIT)	Binding Affinity Energy ΔG (kJ/mol)	Protein-Ligand Complex (LeadIT)	Binding Affinity Energy ΔG (kJ/mol)
5KWW- **JNJ-53718678**	−25.0	5KWW-**22**	−19.0
5KWW-**4**	−3.0	5KWW-**24**	−1.0
5KWW-**19**	−5.0	5KWW-**25**	−20.0
5KWW-**21**	−13.0		

**Table 4:** Absorption, distribution, metabolism and excretion (ADME) descriptors related to absorption and distribution properties.

Comp.	N. H-Bond acceptor	N. H-Bond donor	N. Rotable Bonds	cLogP	HIA (%) ^a^	Vd (l/kg) ^b^	%PPB ^c^	^d^ LogK_a_^HSA^	%F (oral)
**JNJ-53718678**	7	0	8	3.36	100	2.4	99.0	5.42	21.0
**22**	9	3	9	2.81	100	4.1	93.0	3.81	97.0
**25**	8	2	8	3.53	100	7.0	93.0	4.34	99.1

^a^ HIA represents the human intestinal absorption, expressed as percentage of the molecule able to pass through the intestinal membrane; ^b^ prediction of volume of distribution (Vd) of the compound in the body, ^c^ percentage of compound bound to plasmatic protein, ^d^ affinity for albumin.

## Supplementary Materials

The following are available online, Figure S1, Docking positioning of the RSV F protein inhibitor **25** and of the inactive analogue **21**, Figure S2, Docking positioning of the RSV F protein of the inactive analogues **19** and **21**, Figure S3, Docking positioning of the RSV F protein inhibitor **22** and of the inactive analogue **4**.
