# Supplementary material for: Synthesis and Biological Evaluation of Novel (thio)semicarbazone-Based Benzimidazoles as Antiviral Agents against Human Respiratory Viruses
Source: Molecules. 2020 Mar 25;25(7):1487. doi: 10.3390/molecules25071487 (PMC7180491; doi:10.3390/molecules25071487)
Supplement: Supplementary file 1 [file molecules-25-01487-s001.pdf]

Supplementary material

Synthesis and biological evaluation of novel (thio)semicarbazone-based benzimidazoles as antiviral agents against human respiratory viruses

Valeria Francesconi [1], Elena Cichero [1], Silvia Schenone [1], Lieve Naesens [2], and Michele Tonelli [1,*]

[1] Dipartimento di Farmacia, Università di Genova, Viale Benedetto XV 3, 16132 Genova, Italy; francesconi.phd@difar.unige.it (V.F.); cichero@difar.unige.it (E.C.); schenone@difar.unige.it (S.S.)
[2] Rega Institute for Medical Research, KU Leuven, Herestraat 49, B-3000 Leuven, Belgium; lieve.naesens@kuleuven.be (L.N.)
* Correspondence: tonelli@difar.unige.it; Tel.: +39-0103538378

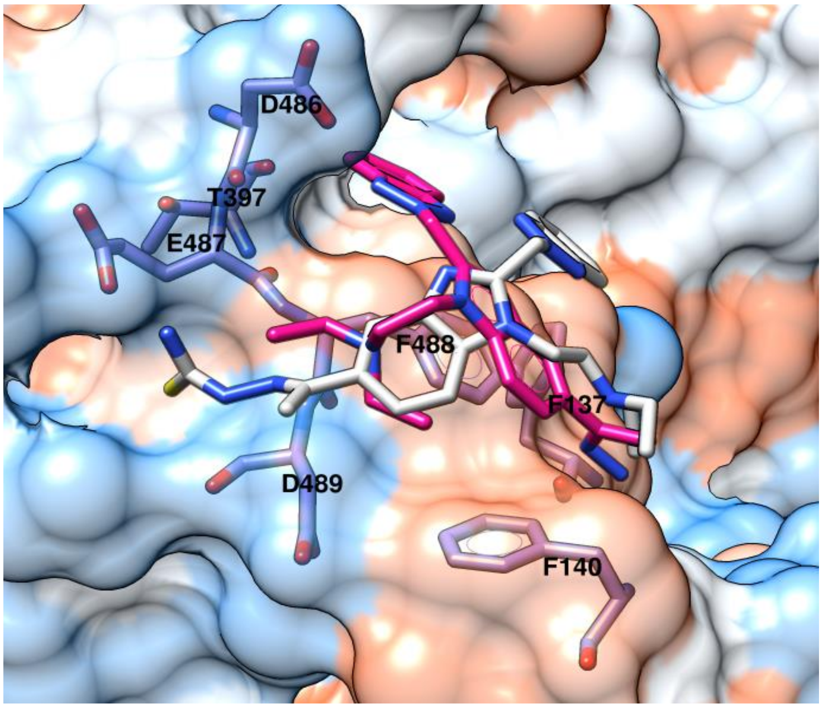

Figure S1. Docking positioning of the RSV F protein inhibitor **25** and of the inactive analogue **21** (*C* atom; deep magenta and white, respectively) are shown within the X-ray crystallographic data of the RSV F protein (pdb code: 5KWW; *C* atom; purple). The most important residues are labelled and coloured by atom type.

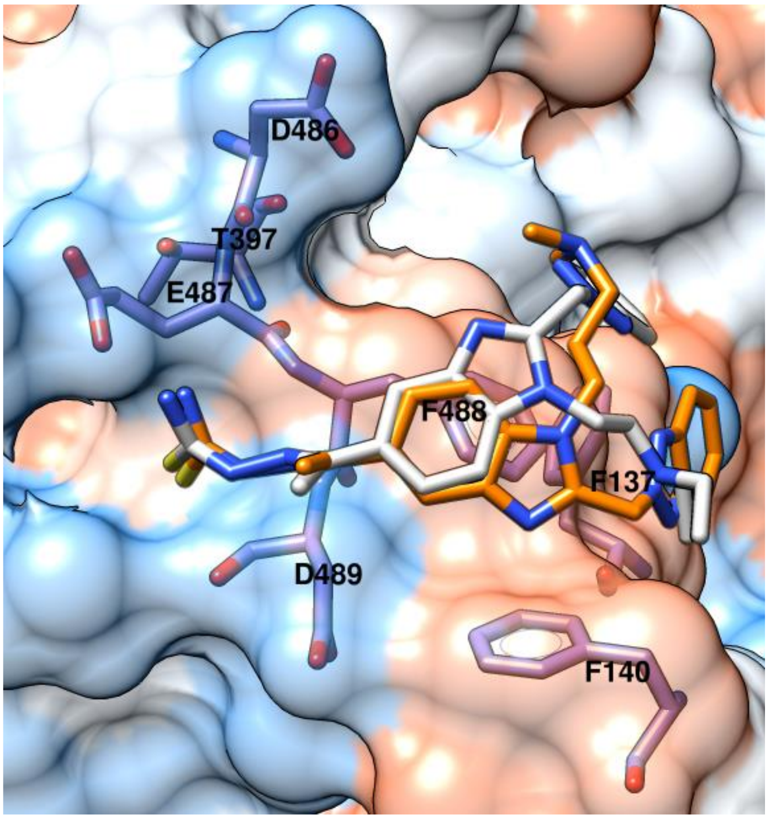

Figure S2. Docking positioning of the RSV F protein of the inactive analogue **19** and **21** (*C* atom; orange and white, respectively) are shown within the X-ray crystallographic data of the RSV F protein (pdb code: 5KWW; *C* atom; purple). The most important residues are labelled and coloured by atom type.

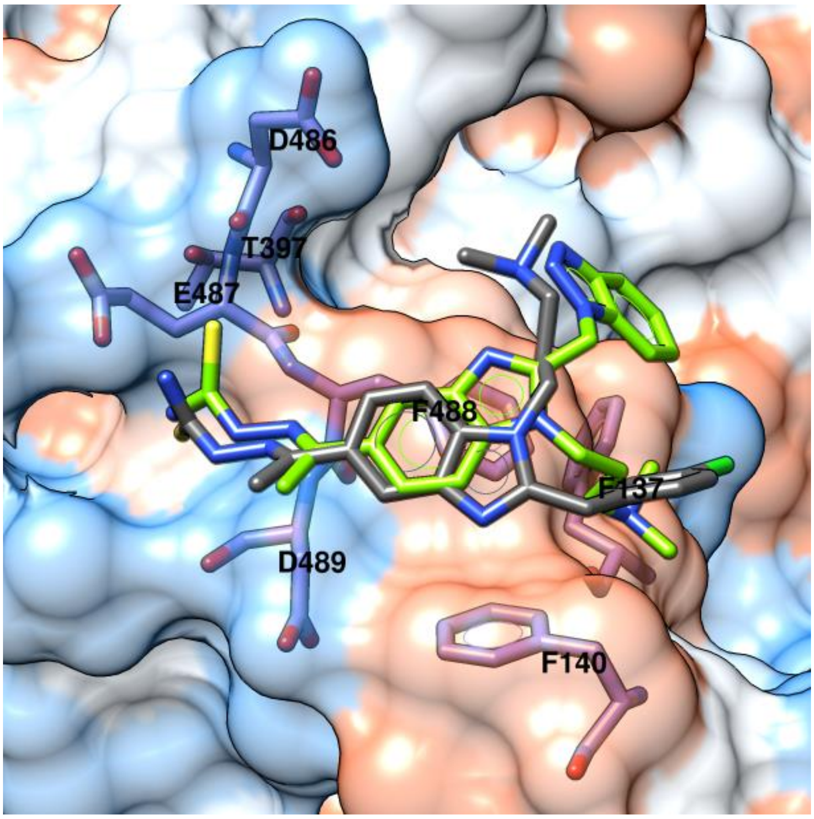

Figure S3. Docking positioning of the RSV F protein inhibitor **22** and of the inactive analogue **4** (*C* atom; green and grey, respectively) are shown within the X-ray crystallographic data of the RSV F protein (pdb code: 5KWW; *C* atom; purple). The most important residues are labelled and coloured by atom type.
